# Spontaneous remission of the micronodular pattern in cryptogenic organizing pneumonia

**DOI:** 10.1002/rcr2.611

**Published:** 2020-06-23

**Authors:** Naoki Kawakami, Rina Kato, Chushu Liu, Masaru Ito, Yoko Wakai, Kazuhito Saito

**Affiliations:** ^1^ Department of Respiratory Medicine Tsuchiura Kyodo General Hospital Ibaraki Japan

**Keywords:** Cryptogenic organizing pneumonia, micronodular pattern, micronodule, spontaneous remission

## Abstract

Organizing pneumonia (OP) is a common interstitial lung disease, pathologically characterized by polypoid granulation tissue in the alveolar ducts and alveoli. In clinical practice, OP occasionally presents as non‐resolving pneumonia. The typical radiographic pattern of OP is characterized by dense consolidation with ground‐glass opacities. Diffuse micronodular pattern of OP (MNOP) is a rare radiographic manifestation that mimics non‐resolving bronchiolar diseases such as pulmonary tuberculosis or hypersensitivity pneumonitis. Steroid therapy is usually effective for MNOP; however, spontaneous remission in MNOP has never been reported. Herein, we report a case of a diffuse micronodular form of cryptogenic OP (COP) that was diagnosed via transbronchial biopsy (TBB) and resolved spontaneously within a few months. Our case highlights that MNOP may resolve spontaneously similar to other forms of OP, and mild cases may be under‐recognized. Furthermore, careful observation could be an option for managing MNOP with mild and non‐progressive symptoms.

## Graphical abstract

Herein, we report a case of a diffuse micronodular form of cryptogenic organizing pneumonia (COP) that was diagnosed via transbronchial biopsy (TBB) and resolved spontaneously within a few months. Our case highlights that diffuse micronodular pattern of OP (MNOP) may resolve spontaneously similar to other forms of OP, and mild cases may be under‐recognized. Furthermore, careful observation could be an option for managing MNOP with mild and non‐progressive symptoms.

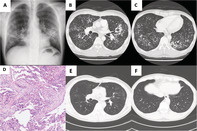

## Introduction

Organizing pneumonia (OP) is a common interstitial lung disease, pathologically defined by the presence of polypoid granulation tissue in the alveolar ducts and alveoli. Clinical manifestations typically include an acute or subacute pneumonic illness characterized by dense consolidation with ground‐glass opacities on radiography [1]. Diffuse micronodular pattern of OP (MNOP) is a rare radiographic manifestation that mimics non‐resolving bronchiolar diseases such as tuberculosis [2]. Steroid therapy is usually effective for MNOP; however, spontaneous remission in MNOP has never been reported [2, 3, 4]. Herein, we report a case of cryptogenic MNOP that resolved spontaneously.

## Case Report

**Figure 1:**
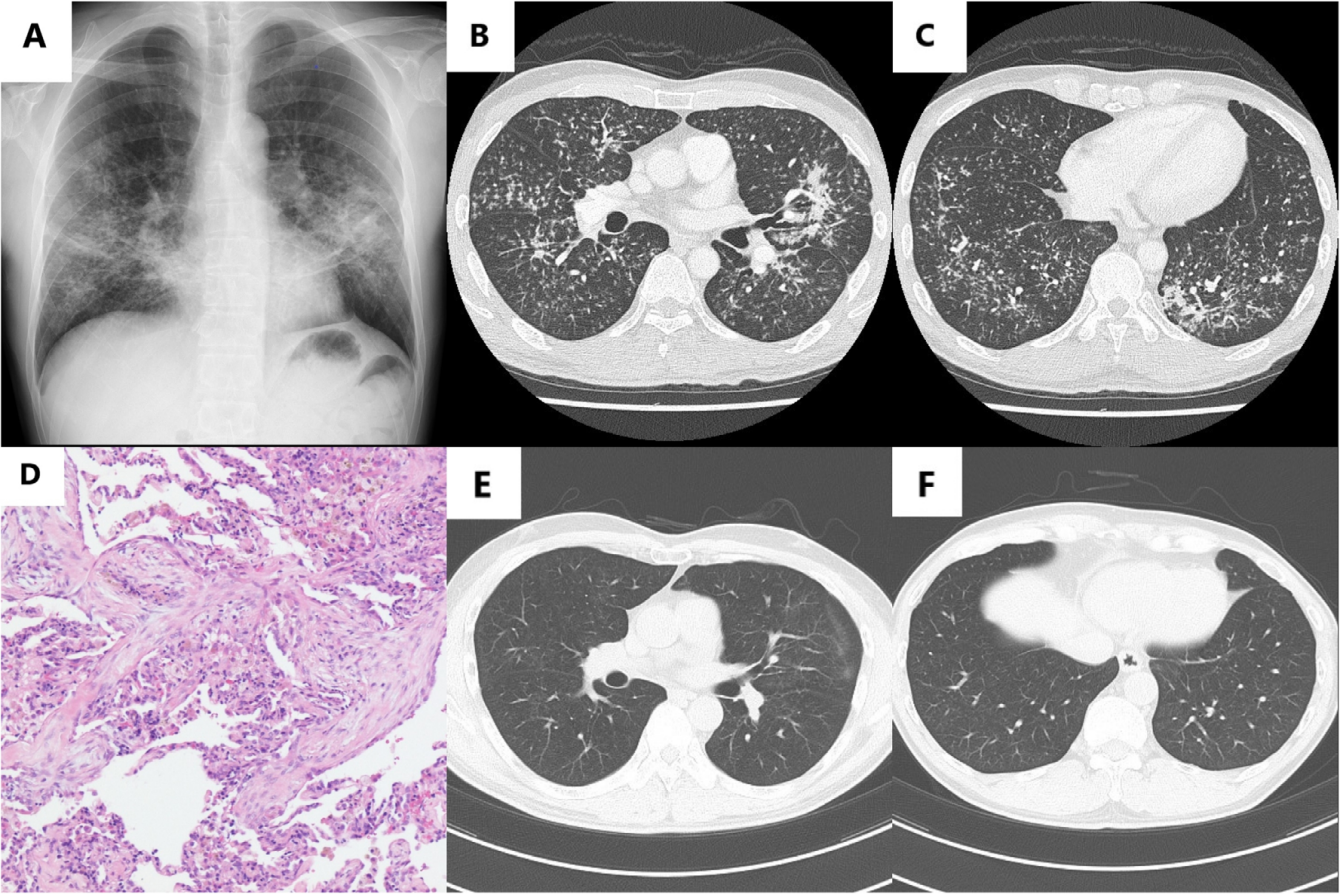
(A) Chest radiograph showing diffuse bilateral micronodules and ill‐defined infiltration. (B, C) Chest computed tomography (CT) scan showing diffuse centrilobular micronodules (<5 mm) and partial consolidation sparing the subpleural area. (D) Pathological findings of a transbronchial biopsy specimen (haematoxylin and eosin staining) showing multiple polypoid granulations in the alveoli and alveolar ducts. No granuloma was observed in any specimen. (E, F) Chest CT scan after three months, showing almost complete resolution of micronodules and consolidation, with very few residual ground‐glass opacities.

A 57‐year‐old man was referred to our hospital with cough and dyspnoea for one month. He was a smoker (30 pack‐years) and also had diabetes. Repeated courses of antibiotics, including fluoroquinolones, were ineffective. He reported no known history of preceding respiratory infection, new medications, or habitual use of inhalants. He was a Buddhist monk and burned incense daily, but had used the same brand of incense for many years. His vital signs and physical examination findings were unremarkable. Spirometry results were normal. Laboratory findings were as follows: white blood cell count of 14,180/μL (83.8% neutrophils), C‐reactive protein of 2.06 mg/dL, lactate dehydrogenase of 150 IU/L, sialylated carbohydrate KL‐6 of 432 U/mL, surfactant protein‐D of 150 ng/mL, and glycated haemoglobin of 7.7%. No findings suggested autoimmune diseases, specific immunodeficiency including HIV infection, or haematological diseases (Table 1). Chest radiography showed diffuse bilateral micronodules and ill‐defined infiltration (Fig. 1A). Chest computed tomography (CT) revealed diffuse centrilobular micronodules (<5 mm) and partial consolidation, sparing the subpleural areas (Fig. 1B, C).

**Table 1:** Peripheral blood test results.

**Haematology**		**Serology and immunology**	
White blood cell	14,180/μL	HBs antigen	Negative
Neutrophils	83.8%	Anti‐HCV antibody	Negative
Lymphocytes	9.6%	HIV screening test	Negative
Monocytes	5.1%	RPR test (quantitative)	Negative
Eosinophils	1.0%	TPHA test (quantitative)	Negative
Basophils	0.5%	(1–3) β‐D glucan	<5 pg/mL
Hemoglobin	15.5 g/dL	Soluble IL‐2 receptor	918 U/mL
Platelet	61.6 × 10^4^/μL	ACE	11.9 IU/L
		KL‐6	432 U/mL
		SP‐D	150 ng/mL
**Biochemistry**		Antinuclear antibody	×80 (homogenous)
Total protein	7.0 g/dL	Anti‐DNA antibody	Negative
Albumin	2.9 g/dL	Anti‐SS‐A antibody	Negative
Blood urea nitrogen	15 mg/dL	Anti‐SS‐B antibody	Negative
Creatinine	0.71 mg/dL	Anti‐RNP antibody	Negative
Uric acid	4.8 mg/dL	Anti‐Sm antibody	Negative
Sodium	139 mEq/L	Anti‐Scl‐70 antibody	Negative
Potassium	4.1 mEq/L	Anti‐ARS antibody	Negative
Calcium	9.3 mg/dL	Rheumatoid factor	<5 IU/mL
AST	33 IU/L	CH50	62.2 U/mL
ALT	48 IU/L	PR3‐ANCA	Negative
LDH	150 IU/L	MPO‐ANCA	Negative
Alkaline phosphatase	482 IU/L	Immunoglobulin G	1555 mg/dL
γ‐GTP	91 IU/L	Immunoglobulin A	268 mg/dL
Total bilirubin	0.4 mg/dL	Immunoglobulin M	77.4 mg/dL
Creatine kinase	32 IU/L	Immunoglobulin E	930.8 IU/mL
Glucose	156 mg/dL		
Glycated hemoglobin	7.7%		
C‐reactive protein	2.06 mg/dL		
Ferritin	223 μg/mL		

AST, aspartate aminotransferase; ALT, alanine aminotransferase; LDH, lactate dehydrogenase; γ‐GTP, γ‐glutamyl transpeptidase; HBs, hepatitis B surface; HCV, hepatitis C virus; HIV, human immunodeficiency virus; RPR, rapid plasma regain; TPHA, treponema pallidum haemagglutination; IL, interleukin; ACE, angiotensin converting enzyme; KL‐6, sialylated carbohydrate antigen KL‐6; SP‐D, surfactant protein D; ARS, aminoacyl transfer RNA synthetase; CH50, 50% hemolytic complement activity; PR3, proteinase 3; MPO, myeloperoxidase; ANCA, anti‐neutrophil cytoplasmic antibody.

Bronchoalveolar lavage fluid (BALF) collected through the right B5a showed a lymphocyte‐dominant pattern (38% lymphocytes, 7% neutrophils, 2% eosinophils, and 53% macrophages), no atypical cells, and a 0.32 CD4/CD8 ratio. Transbronchial biopsy (TBB), performed through the right B4a and right B8a, revealed numerous polypoid granulations in the air spaces, and no granuloma was observed (Fig. 1D). Microbiological tests for bacteria, mycobacteria, and fungi using BALF and biopsy specimens were negative. We did not perform molecular biological testing for infectious pathogens using BALF, as the remaining BALF was discarded. A diagnosis of cryptogenic MNOP was made.

The patient's symptoms had already improved when the TBB results were received. We did not administer corticosteroids, and the patient was kept under careful observation because of mild and spontaneously abating symptoms and the risk of worsening diabetes. His symptoms abated within two weeks and radiographic findings resolved gradually and spontaneously over three months (Fig. 1D, E). Relapse was not observed for three years after the first presentation.

## Discussion

Typical manifestations of OP/cryptogenic OP (COP) are well known: acute or subacute pneumonic illness accompanied by patchy air space consolidation with a migratory course and peripheral ground‐glass opacities on CT [1].

MNOP is a relatively rare radiographic manifestation observed in up to 24% of OP cases. It is prevalent in immunocompromised patients [2]. In MNOP, symptoms including fever, cough, and dyspnoea are similar to those with other radiographic patterns of OP [2].

In our case, no obvious cause or associated comorbidity was found except for moderate diabetes. Conditions associated with MNOP are substance abuse (marijuana), haematological malignancies, solid organ transplantation, infections, and immune checkpoint inhibitor use [2, 3, 4]. It is unknown whether diabetes is associated with MNOP. Regarding the reported cases, similar to other OP forms, histopathological findings could not distinguish between cryptogenic and secondary MNOP. Moreover, clinical and radiographic features in cryptogenic and secondary MNOP are similar [2, 3]. Therefore, diagnosis of cryptogenic MNOP is made by a multidisciplinary evaluation in the same manner as for typical COP.

In this case, we suspected tuberculosis at the first presentation because of the symptoms and radiographic findings. Typical CT findings of MNOP include micronodules, often with centrilobular distribution, that are indistinguishable from bronchiolar diseases such as bronchopneumonia, tuberculosis, and hypersensitivity pneumonitis [2].

Our patient's BALF was lymphocytic, and this is typical for OP [1]. However, it is unknown whether the BALF findings in MNOP are similar to those in other OP forms because they have rarely been mentioned in the reported MNOP cases.

Our case was successfully diagnosed via TBB. TBB or surgical lung biopsy provides a definitive pathological diagnosis of MNOP [2, 3]. Only around 30% of reported MNOP cases were diagnosed via TBB; however, the sensitivity of TBB is unknown, because only a subset of patients underwent TBB [2]. In our case, we performed TBB using a guide sheath to avoid excessive bleeding from repeated tissue sampling through the same bronchus. This approach might be effective for sampling enough tissue to diagnose MNOP. Cryobiopsy may be a promising procedure in the diagnosis of MNOP, as it is used to diagnose other types of interstitial lung diseases [5].

As in other forms of OP, corticosteroids rapidly achieve symptomatic and radiographic resolution of both cryptogenic and secondary MNOP [2, 3, 4]. It was reported that approximately 50% of typical COP cases spontaneously resolved [1], whereas all the reported MNOP cases required corticosteroid treatment because of the acute symptoms, and only one fatality was reported [2, 3, 4]. To our knowledge, this is the first documented case of MNOP showing spontaneous remission. Our case suggests that mild MNOP can spontaneously resolve similar to typical COP, and therefore, mild cases may be under‐recognized. Moreover, although relapse is common in COP, none have been reported for MNOP, including the present case [2].

In conclusion, MNOP is an unusual but important differential diagnosis in patients with diffuse micronodular pulmonary opacities, and MNOP may resolve spontaneously similar to other forms of OP. Careful observation could be an option for managing MNOP with mild and non‐progressive symptoms.

### Disclosure Statement

Appropriate written informed consent was obtained for publication of this case report and accompanying images.
